# The ethical challenges of antimicrobial resistance for Nurse practitioners

**DOI:** 10.1002/nop2.453

**Published:** 2020-01-22

**Authors:** Sarah Oerther, Daniel B. Oerther

**Affiliations:** ^1^ School of Nursing Saint Louis University St. Louis Missouri; ^2^ Fulbright Visiting Scholar King's College London London UK

Antimicrobial resistance (AMR) is one of the most challenging worldwide health threats facing modern medicine (Centers for Disease Control, 2018; Logan & Bonomo, 2016; World Health Organization, 2018; Zerr et al., 2014). According to the World Health Organization (2018), when antimicrobial drugs are no longer effective at killing infections caused by bacteria, fungi, parasites and viruses, this is called AMR. AMR leads to drug‐resistant infections (World Health Organization, 2018). Nurse practitioners need to be leaders in mitigating risks associated with antimicrobial use, including ethical dilemmas surrounding AMR (Centers for Disease Control, 2018; Logan & Bonomo, 2016; World Health Organization, 2018; Zerr et al., 2014). For instance, prescribing antimicrobial drugs represents an ethical dilemma for nurse practitioners, since the health needs of individual patients must to be balanced against preservation of effective antimicrobial therapies and concerns for long‐term prevention of AMR in communities (Basu & Garg, 2018; Johnstone, 2016).

## 1 CURRENT CONSIDERATIONS FOR PRIMARY CARE NURSE PRACTITIONERS

The prognosis of AMR is increased morbidity and mortality from uncontrolled growth of a microorganism that is resistant to one, or even all, available antimicrobial therapies (Centers for Disease Control, 2018; Logan & Bonomo, 2016; World Health Organization, 2018; Zerr et al., 2014). In hospital environments, microorganisms that are resistant to antimicrobial therapies lead to persistent, pervasive nosocomial infections that may be highly contagious, nearly impossible to treat and possibly lethal (Centers for Disease Control, 2018; World Health Organization, 2018; Zerr et al., 2014). To provide quality care for patients with AMR, three practices are critical (Centers for Disease Control, 2018; Logan & Bonomo, 2016; World Health Organization, 2018; Zerr et al., 2014). First, hospitals and outpatient clinical facilities must be (re)designed to prevent the spread of nosocomial infections. Second, additional rapid bedside diagnostics are required to evaluate antimicrobial susceptibility in real‐time as part of the decision‐making process in prescribing an antimicrobial therapy. Third, more expensive, last‐line‐of‐defence antimicrobials will need to be reserved for longer inpatient treatment scenarios (Centers for Disease Control, 2018; Logan & Bonomo, 2016; World Health Organization, 2018; Zerr et al., 2014).

In the long‐term, the threat from a postantimicrobial therapy world is that today's relatively minor infections—including practically unavoidable exposures to microbes associated with labour and delivery—may once again contribute to increased morbidity and mortality in both developed and in developing countries (Centers for Disease Control, 2018; Logan & Bonomo, 2016; World Health Organization, 2018; Zerr et al., 2014). In developed countries, unchecked AMR may impact surgery and chemotherapy because antibiotics used to treat infections may no longer be effective (Centers for Disease Control, 2018; Logan & Bonomo, 2016; World Health Organization, 2018; Zerr et al., 2014). And in developing countries, unchecked AMR may also co‐occur alongside and impact the incidence and prevalence of existing wide‐spread infections such as HIV, malaria and tuberculosis (Centers for Disease Control, 2018; World Health Organization, 2018).

Currently, nurse practitioners are unable to easily and quickly screen and diagnose many types of AMR because specimens must be sent to a centralized diagnostic laboratory for evaluation of antimicrobial susceptibility that often takes several days of testing (Mitsakakis, Kaman, Elshout, Specht, & Hays, 2018). Until better bedside diagnostics are available, one of the best means of preventing the spread of AMR by nurse practitioners may be accomplished through a thorough history and physical exam, and by screenings (Ball, Dains, Flynn, Solomon, & Stewart, 2019; Hersh, Jackson, & Hicks, 2013). For instance, final diagnosis of a viral upper respiratory illness can usually be made clinically with little or no laboratory testing needed (Ball et al., 2019; Hersh et al., 2013). However, if a pre‐school age child exhibits a high fever with sudden onset, body aches and fatigue, a primary care provider might use other screenings to rule‐out a specific alternative differential diagnosis, such as influenza A or B (Ball et al., 2019; Hersh et al., 2013). Conversely, if a child presents with a severe sore throat, without a cough or other upper respiratory symptoms, it may be appropriate to do a rapid strep test to rule‐out group A streptococcus infection (Ball et al., 2019; Muthanna, Salim, Hamat, Shamsuddin, & Zakariah, 2018). Unfortunately, there is no test to diagnose an upper respiratory tract infection in an outpatient clinical setting (Ball et al., 2019). Similarly, the diagnosis for bacterial sinusitis, acute otitis media or viral bronchitis must be made via clinical examination, without the support of diagnostic testing (Ball et al., 2019).

## 2 ETHICS

AMR needs to be viewed by nurse practitioners not just as a primary care issue but also an ethical issue (Basu & Garg, 2018; Johnstone, 2016). Nurse practitioners face three ethical challenges associated with AMR. First, the nurse practitioner must identify the safest ways to protect and promote the individual patient's well‐being in the face of the collective threat to human health represented by AMR. Second, the nurse practitioner must identify how best to respond to competing needs as the amount of AMR increases and the effectiveness of existing antimicrobial drugs continues to decrease. Third, the nurse practitioner must balance the moral interest of different people, including future generations, while deciding and justifying a course of action that “normally” would not be morally acceptable (e.g. refusing to prescribe an antimicrobial therapy and relying upon the expectation that an infection is self limiting). These ethical questions also bring up important concerns about the sufficiency and suitability of current ethical frameworks to tackle concerns surrounding AMR (Basu & Garg, 2018; Johnstone, 2016).

An important distinctive feature of AMR is the collective and reciprocal vulnerability of all people both as the vectors and victims of AMR organisms (Basu & Garg, 2018; Johnstone, 2016). Nurse practitioners must advocate in healthcare institutions that patients are not just stand‐alone discrete individuals—a balance must be found between the individual patient and the collective health of the community. For instance, some hospitals are trying to stop the spread of AMR by encouraging healthcare providers to prescribe narrow‐spectrum antibiotics while reserving broad‐spectrum drugs for emergencies or last resort (Ellegård, Dietrichson, & Anell, 2017). Ellegård et al. (2017) examined pay for performance (P4P)—monetary incentives to reach pre‐defined prescribing targets—as a way to influence prescribers’ choice of antibiotics. Data from eight Swedish healthcare authorities used the P4P model as a way to encourage prescribers to select narrow‐spectrum antibiotics more often in the treatment of children with respiratory tract infections (Ellegård et al., 2017). They found P4P significantly increased the use of narrow‐spectrum antibiotics (Ellegård et al., 2017).

Unfortunately, the combination of monetary incentives from some hospitals a few different countries and limited knowledge about prescription guidelines for antimicrobial therapies can contribute to unethical prescribing practices (Basu & Garg, 2018; Johnstone, 2016). Additionally, monetary prescribing practices may violate ethical principles core to health care, including: beneficence, patient autonomy, non‐maleficence and justice (Basu & Garg, 2018). This incentive system may contribute to violation in the ethical principle of justice, which requires fair distribution of limited resources (Basu & Garg, 2018). A nurse practitioner with prescription authority, anticipating ineffectiveness of a narrow‐spectrum antibiotic and concerned with the risk of prolonged sickness among influential patients, may elect to prescribe a broad‐spectrum antibiotic to those whom they favour (Basu & Garg, 2018). While such differential treatment is unethical and could contribute to class bias, it is not beyond reason to foresee a perverse outcome arising from such a monetary incentive with the quality of medical care distributed based upon the resources of patients (Basu & Garg, 2018; Johnstone, 2016). Therefore, nurse practitioners need to be on‐guard when well‐intentioned policies to prevent AMR may lead to unanticipated outcomes of questionable ethical practice.

## 3 CONCLUSION

AMR brings to the forefront ethical issue that requires a new way of thinking for nurse practitioners, not just about the nature of the ethical issues AMR raises, but about how to best address these moral dilemmas (Johnstone, 2016). The imperatives of tackling the gaps in healthcare ethics related to AMR can no longer be ignored by nurse practitioners. New innovative systems and theoretical frameworks are urgently needed to address the moral issues raised by AMR (Basu & Garg, 2018; Johnstone, 2016). As the threat from AMR grows, innovative systems and theoretical frameworks will have a major influence on how the above questions are addressed. The answers to the above questions will have a major impact on the types of changes that eventually will need to be made to current standards of care. In a postantimicrobial era, these will describe what counts as “optimal care.” What future standard of optimal care will look like is unclear. As a vital part of the primary care workforce, nurse practitioners need to start preparing now through collaborative inquiry and education for the ethical challenges that remain ahead in guaranteeing just, humane and socially responsible consideration to the management and prevention of AMR (Johnstone, 2016).

## AUTHOR CONTRIBUTIONS

All authors contributed equally to this article.
